# 在基因序列中高效引入多位点突变的方法

**DOI:** 10.3779/j.issn.1009-3419.2014.06.06

**Published:** 2014-06-20

**Authors:** 凡荣 孟, 琛 陈, 永文 李, 海粟 万, 清华 周

**Affiliations:** 300052 天津，天津医科大学总医院，天津市肺癌研究所，天津市肺癌转移与肿瘤微环境实验室 Tianjin Key Laboratory of Lung Cancer Metastasis and Tumor Microenviroment, Tianjin Lung Cancer Institute, Tianjin Medical University General Hospital, Tianjin 300052, China

**Keywords:** 多位点突变, 突变引物, Type Ⅱs类的限制性内切酶, Multiple-site mutagenesis, Mutagenic primers, Type Ⅱs restriction enzyme

## Abstract

**背景与目的:**

在基因序列中引入点突变是研究基因结构和功能及其相关性的重要手段。目前已有多种对基因序列进行突变的方法，然而，这些方法大多对单一位置的基因突变有效，而对在基因序列中引入多位点突变，还有待方法的进一步改进。为适应这一需要，本研究提供了一种高效的可在基因序列的多个位点引入突变的方法。

**方法:**

该方法依赖于一种Type Ⅱs类的限制性内切酶，例如*Esp* 3I等。本研究所提供的方法中，针对每一个突变位点，合成一对含有突变点和所选择的Type Ⅱs类的限制性内切酶位点的引物，当需要引入多位点突变时，则利用邻近的两个突变位点的引物做PCR反应，多个位点的突变，就可以得到多个扩增片段，将这些片段用和引物上的限制性内切酶位点对应的酶进行反应，然后用连接反应将片段连接形成突变基因。

**结果:**

本研究所提供的方法非常简便，主要实验步骤可以在一天之内完成。我们已经利用这种方法，在绿色荧光蛋白（enhanced green fluorecence protein, *EGFP*）和*nm23*基因中，引入3个或4个突变，突变效率几乎为100%。

**结论:**

本研究所提供的方法可以成为研究基因功能的有用工具。

## Abstract (en)

**Background and objective:**

The methods for introducing point mutations into target genes are important for dissecting the relationship of gene structure and function. Up to date, there are numbers of protocols available for the purpose. However, many of them are suited for introducing single site mutation into the target gene. For introducing multiple-site mutations simultaneously into the target genes, it is required to further improve the related methods.

**Methods:**

In this report, we describe an improvement on the type Ⅱs restriction enzyme-dependent site-directed mutagenesis method and the improved protocol is highly efficient for multiple-site mutagenesis. In our method, a pair of mutagenic primers are synthesized for each desired site and each mutagenic primer contains a selected type Ⅱs restriction site. The DNA fragments between two neighboring sites are amplified with PCR. All amplified fragments are then digested by the selected Type Ⅱs restriction enzyme. The expected mutant is eventually generated by ligation of these digested DNA fragments.

**Results:**

The improved protocol is very easy and can be achieved in just one day. As a proof of principle, we have introduced multiple-site, *i.e.*, 3-site or 4-site mutations into the fusion gene of *nm23* and *EGFP* (enhanced green fluorecence protein). The mutagenic frequencies are almost reached 100%.

**Conclusion:**

Our protocol provides a useful tool for gene function research.

在基因序列中引入点突变，是研究基因的结构和功能及其相关性的重要手段^[1, 2]^。例如，当所研究的基因是蛋白编码基因时，在蛋白编码框架内引入点突变，就可以改变蛋白编码序列，而突变基因在相关研究体系中功能的变化，则可以为该基因的功能特点的研究提供依据。体外基因突变已经成为分子生物学实验室的一项常规工作。为适应这一需要，已经有多种在基因序列中引入点突变的方法可以使用^[3-5]^。目前最常用的方法Quickchange^TM^占据了主流的位置，当然，也存在其它许多可以实现基因定点突变的方法，但这些方法中，基本都是对在单一位置引入点突变较为有效，而对在基因序列的多个位点引入突变则并不有效。为解决这一问题，有Ko等^[6]^提供了一种可以在体外基因序列中引入多个点突变的方法，但这种方法含有琼脂糖凝胶电泳的步骤，以及其后的DNA片段的回收，操作过程极为繁琐，因此，并不太适合作为实验的常规方法使用。本研究在这些方法的基础上，提供一种非常简便有效的在基因序列中引入多位点突变的方法，本研究所提供的方法主要实验步骤可在一天之内完成，突变效率可接近100%^[7-9]^。

## 1 材料与方法

### 1.1 材料

本研究使用的基因序列是pEN载体，其中含有nm23和绿色荧光蛋白（enhanced green fluorecence protein, EGFP）之间的融合基因，该载体为实验室常规保存。PCR反应所用试剂盒PrimeSTAR HS DNA Polymerase（DR010A），T4DNA连接酶（T4 DNA ligase, D2011），DNA纯化DNA Fragment Purification Kit（DV807A, TaKaRa）及DH5alpha菌株为宿主，均购自Takara（大连）公司。限制性内切酶*Esp* 3I及*Dpn* I为Fermentas产品。质粒小提试剂盒购自天根生化科技（北京）有限公司。

### 1.2 方法

#### 1.2.1 引物合成

在一段位于pEN载体的基因序列中引入多位点的定点突变时，针对每一个突变位点，合成一对引物，待突变的位点则位于该引物序列之中，而每一个引物中均含有限制性内切酶*Esp* 3I位点。则每个扩增片段的两端都含有一个*Esp* 3I位点。实验所用的突变引物由Takara合成，具体序列见表 1。

**1:** 实验所使用的突变引物序列 Oligonucleotides used in the experiments

Site	Sequences 5'-3'
Site1A	5'-gatgacgtctca 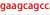 atggccaactgtgagcgtaccttcattgcgatca-3’
Site1B	5'-gcttaaccgtctctcttccttgagctcgagatctgagtccggtag-3’
Site2A	5'-gaacacgtctcaattc 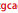 cttgttggtctgaaattcatgcaagcttccg-3’
Site2B	5'-gataacgtctctgaatcctttctgctcaaaacgcttgataatctctc-3’
Site3A	5'-gaagacgtctcac 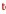 ctgcagactccaagcctgggaccatccg-3’
Site3B	5'-gaaatcgtctcaagagagcatgactcggcccgtcttcaccacattcag-3’
Site4A	5'-gcgatcgtctct 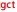 tttcaccctgaggaactggtagattacacgagc-3’
Site4B	5'-gtttccgtctccaagccaagccgatctccttctctgcactctc-3’
The type Ⅱs restriction enzyme site, *Esp*3I, is underlined. The mutational sites are in red.	

#### 1.2.2 PCR产物制备

PCR反应利用PrimeSTAR HS DNA Polymerase及其中的反应试剂（DR010A, Takara）进行。反应体系为100 µL，含有以下成分：1×Prime STAR buffer；dNTP Mixture，其中dATP、dGTP、dCTP、dTTP的终浓度均0.25 nM，Primer A、Primer B的终浓度均为0.25 µM，质粒DNA（pEN载体）20 ng，PrimeSTAR HS DNA Polymerase 2.5 U。PCR反应循环条件为：预变性95 ℃、30 s后进入循环，98 ℃、10 s变性，62.5 ℃、30 s退火，72 ℃、5 min复性，共28个循环，继续72 ℃延伸10 min后终止反应，产物4 ℃保存备用。

#### 1.2.3 模板质粒DNA的去除

将上一步骤的PCR产物转入37 ℃水浴，直接加入限制性内切酶*Dpn*I 40 U（Fermentas）酶切1 h-2 h。对所得DNA产物进行纯化，所用试剂盒为DNA Fragment Purification Kit（DV807A, TaKaRa），30 µL水复溶。

#### 1.2.4 连接末端的形成及连接反应

对扩增产物用*Esp* 3I对其进行酶切，从而将引物序列中非特异的序列部分去除并形成连接末端，具体反应条件为：将以上纯化后的扩增产物放入50 µL酶切体系中，其中含有*Esp* 3I量为20 U（Fermentas），其它条件与厂家要求一致，37 ℃水浴2 h。实验接着对所得酶切产物进行纯化，所用试剂盒为DNA Fragment Purification Kit（DV807A, TaKaRa）。最后，所得DNA纯化产物进行连接反应（T4 DNA Ligase, Takara），16 ℃水浴1 h-2 h。

#### 1.2.5 化学转化DH5alpha感受态细胞

取所得连接产物1 µL，转入100 µL感受态细胞混匀，冰上30 min，42 ℃水浴1 min，然后将受体菌加入到预热至37 ℃未加抗生素的LB培养基悬浮，37 ℃摇床培养1 h，200 rpm/min。涂布于含卡那霉素的LB平板，37 ℃培养过夜后挑单菌落鉴定质粒。

#### 1.2.6 突变质粒的鉴定和基因序列的测定

质粒小提试剂盒提取质粒，含有突变基因的质粒，利用Sanger测序法进行测序验证（华大基因科技服务有限公司，北京）。

## 2 结果

### 2.1 在基因序列中引入多位点突变的流程

在一个位于某个表达载体的基因序列中引入多位点的定点突变时，针对每一个突变位点合成一对引物，待突变的位点则位于该序列之中，而每一个引物中均含有一个合适的Type Ⅱs类的限制性内切酶的位点，一般情况下，我们使用*Esp* 3I位点。当然，也有许多其它的该类型的限制性内切酶位点可以使用。这一类限制性内切酶的特点是，其在底物DNA序列上的识别位点与其在底物序列上的酶切位点是分离的^[10]^。常用的*Esp* 3I限制性内切酶的识别位点和酶切位点被置于突变引物序列的5’端，而突变位点和周围的特异序列，则被置于突变引物序列的3’端。如图 1所示，实验首先利用常规的PCR反应，对邻近两个突变位点之间的序列进行扩增，假定需要在基因序列中引入4个位点的突变，则可以将载体分别扩增为4个片段，其中，每个扩增片段的两端都含有一个*Esp* 3I位点，然后借助*Dpn* I限制性内切酶，对扩增产物进行酶切，以消除作为扩增模板的质粒，防止其在后续的实验中形成假阳性克隆^[11]^。实验接着利用对应的Type Ⅱs类的限制性内切酶，对扩增产物进行酶切，从而将引物序列中非特异的序列部分去除并形成连接末端，然后，借助T4 DNA连接酶做连接反应，以形成突变载体。和其它类似的方法比较，我们这里提供的方法中，没有任何DNA电泳和回收的步骤，因此，在实验操作时非常简便易行。

**1:**
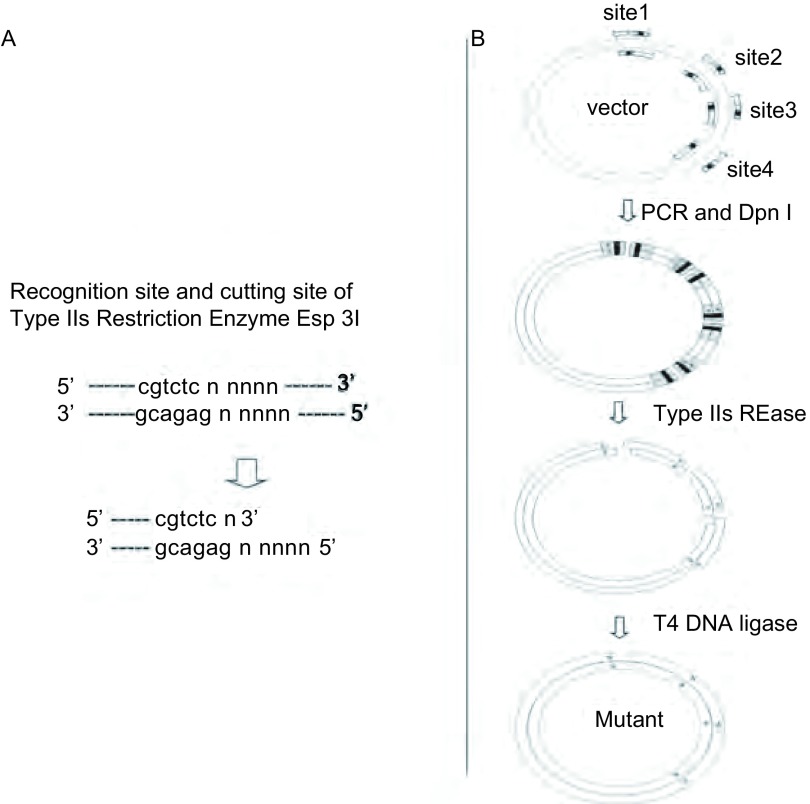
*Esp* 3I酶切位点及实验流程示意图 The recognition sequence of *Esp* 3I and the schematic representation of the method

### 2.2 在*nm23-EGFP*融合基因序列中引入3个和4个位点的突变

为验证以上实验方法是否可行，我们利用在pEN载体中含有的*nm23-EGFP*融合基因中引入3个-4个突变来进行验证^[12]^。如图 2所示，实验共选择在4个位点，分别用下划线标注，被称为突变位点site1、site2、site3、site4。每个位点对应的突变引物分别是：site1A1B、site2A2B、site3A3B、site4A4B。每个引物的5’端含有一个属于Type Ⅱs类限制性内切酶的*Esp* 3I位点。这些突变有的属于难度较大的突变类型，因此可以验证本研究所提供的方法的有效性。

**2:**
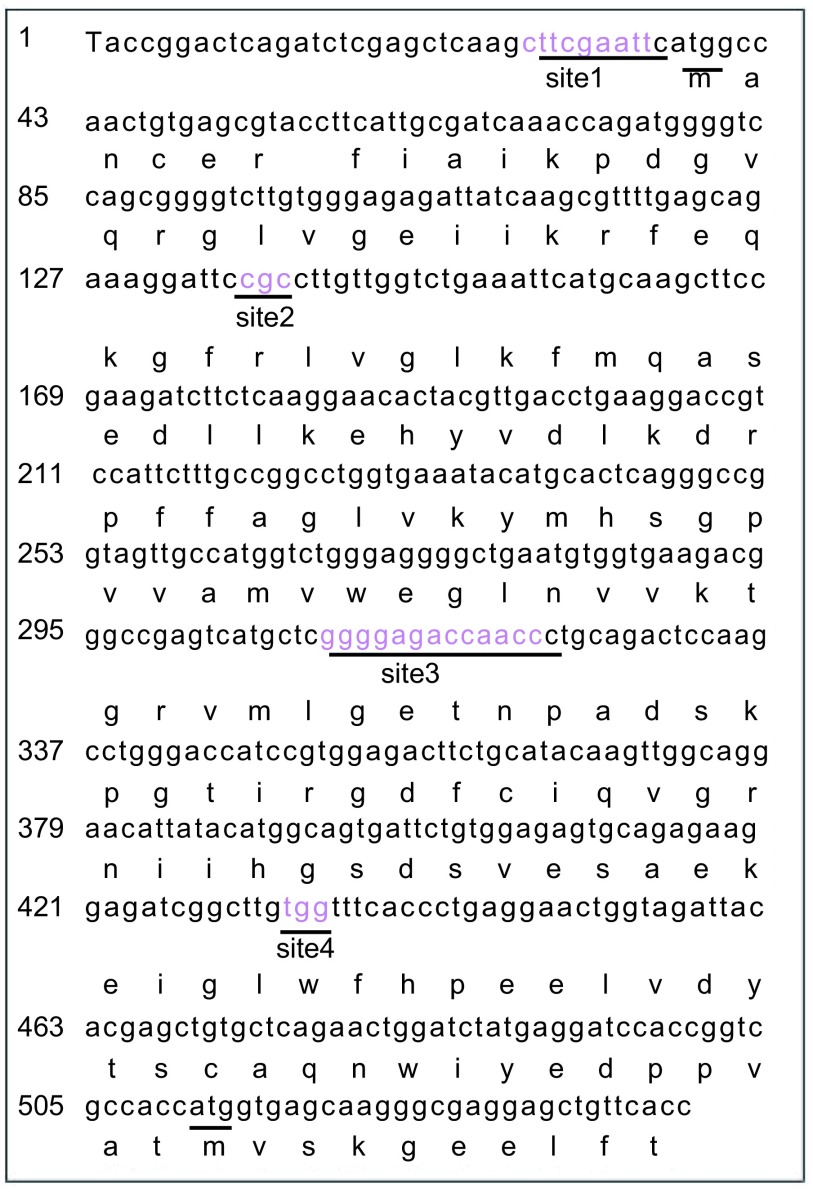
*nm23*-*EGFP*融合基因序列中的突变位点 Sequence of the *nm23*-*EGFP* fusion gene and the desired mutagenic sites

在这个实验中，如果引入3个位点的突变，则有4种组合，即123、124、234、134，而4个位点的突变只有一种，即1234。实验对所有这些组合进行了测试，它们分别被称为突变123、124、234、134、1234。实验分别利用两个邻近的位点引物做PCR扩增，对于需要引入3个位点的突变的情形，先扩增出3个片段，而对于需要引入4个位点突变的情形，共需要扩增出4个片段。然后，实验利用*Esp* 3I对其进行酶切，并借助连接反应将酶切后片段连接在一起，形成所需要的突变载体。实验利用基因测序的方法对所获得突变载体克隆进行了验证，如图 3A所示，突变123、124、234、134、1234的成功率分别为：7/7（100%）、10/10（100%）、8/9（89%）、9/9（100%）、12/12（100%），成功率接近100%，说明方法的有效性。图 3B中所示为四个突变位点突变后碱基，其中，突变位点site1为9 bp长度的置换突变，突变位点site2为一个氨基酸编码序列的改变，突变位点site3为13bp的序列删除，突变位点site4为单氨基酸编码转换。突变后序列在所设计引物中均有所体现，在表 1中用黑体字标出。实验测序结果充分验证了方法的有效性。

**3:**
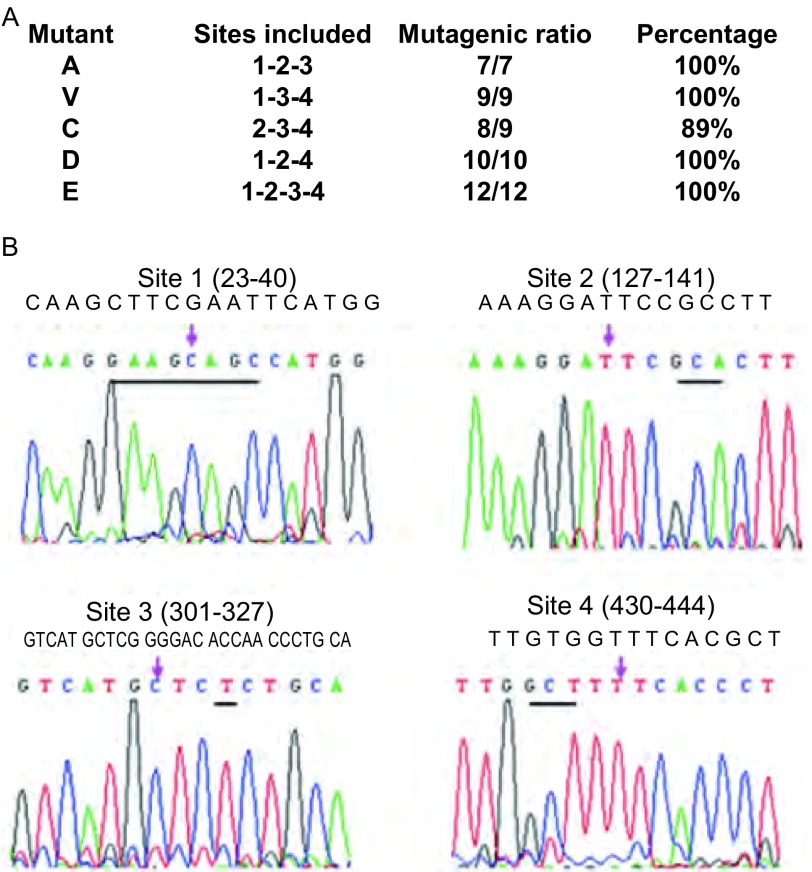
实验突变结果及测序图。A：突变1-2-3、1-2-4、2-3-4、1-3-4和1-2-3-4的突变成率；B：四个位点的突变测序图。 Sequencing results of the mutants. A: mutational rate of the mutants; B: sequencing maps of the mutants.

## 3 讨论

体外基因定点突变技术，已经是分子生物学实验中的一个常用技术，但多数相关的实验方法，只适用于单一位点的序列突变，而对于在目标基因中的多个位点引入突变，实验程序则非常繁琐。本研究所提供的实验方法，是在既有的依赖Type Ⅱs类的限制性内切酶的方法的基础上形成的。Ko等^[6]^报道了一个可用于多位点突变的方法，但这种方法需要凝胶电泳和DNA回收等步骤，很难在短时间内完成，因此并不适合做为一般的实验方法使用，尤其是那些需要对多个样品进行突变的情况。本研究所提供的方法中，则完全避免了凝胶电泳和DNA回收等步骤，实验程序简单，而且可在短时间内完成，也适用于同时处理多个样品的情况。在本研究提供的实验程序中，我们一般会在实验的前一天，做第一步的PCR反应，这样，在次日正式开始实验时，就可以进行后续的酶切和纯化步骤，后面的实验步骤就可以于一天内完成。为了验证实验程序的有效性，我们在*nm23-EGFP*融合基因中，分别引入了三个或者四个位点的突变，实验结果都获得了接近100%的突变率。本研究所提供的方法，为基因结构和功能的研究提供了一个有用的工具。
